# A Novel Synbiotic Alleviates Autoimmune Hepatitis by Modulating the Gut Microbiota-Liver Axis and Inhibiting the Hepatic TLR4/NF-κB/NLRP3 Signaling Pathway

**DOI:** 10.1128/msystems.01127-22

**Published:** 2023-02-16

**Authors:** Yongbo Kang, Xiaoyu Kuang, Huan Yan, Peng Ren, Xiaodan Yang, Haixia Liu, Qingqing Liu, Hao Yang, Xing Kang, Xiaorong Shen, Mingwei Tong, Lin Li, Xiaohui Wang, Linzhi Guo, Jieqiong Ma, Fan Zhang, Weiping Fan

**Affiliations:** a Department of Microbiology and Immunology, School of Basic Medical Sciences, Shanxi Medical University, Taiyuan, Shanxi, China; b Laboratory of Morphology, Shanxi Medical University, Taiyuan, Shanxi, China; Vall d'Hebron Institut de Recerca

**Keywords:** novel synbiotic, autoimmune hepatitis, intestinal permeability, intestinal flora, pyroptosis

## Abstract

Autoimmune hepatitis (AIH) is a liver disease characterized by chronic liver inflammation. The intestinal barrier and microbiome play critical roles in AIH progression. AIH treatment remains challenging because first-line drugs have limited efficacy and many side effects. Thus, there is growing interest in developing synbiotic therapies. This study investigated the effects of a novel synbiotic in an AIH mouse model. We found that this synbiotic (Syn) ameliorated liver injury and improved liver function by reducing hepatic inflammation and pyroptosis. The Syn reversed gut dysbiosis, as indicated by an increase in beneficial bacteria (e.g., *Rikenella* and *Alistipes*) and a decrease in potentially harmful bacteria (e.g., Escherichia*-Shigella*) and lipopolysaccharide (LPS)-bearing Gram-negative bacterial levels. The Syn maintained intestinal barrier integrity, reduced LPS, and inhibited the TLR4/NF-κB and NLRP3/Caspase-1 signaling pathway. In addition, microbiome phenotype prediction by BugBase and bacterial functional potential prediction using Phylogenetic Investigation of Communities by Reconstruction of Unobserved States (PICRUSt) showed that Syn improved gut microbiota function involving inflammatory injury, metabolism, immune response, and pathopoiesia. Furthermore, the new Syn was as effective as prednisone against AIH. Therefore, this novel Syn could be a candidate drug for alleviating AIH through its anti-inflammatory and antipyroptosis properties that relieve endothelial dysfunction and gut dysbiosis.

**IMPORTANCE** Synbiotics can ameliorate liver injury and improve liver function by reducing hepatic inflammation and pyroptosis. Our data indicate that our new Syn not only reverses gut dysbiosis by increasing beneficial bacteria and decreasing lipopolysaccharide (LPS)-bearing Gram-negative bacteria but also maintains intestinal barrier integrity. Thus, its mechanism might be associated with modulating gut microbiota composition and intestinal barrier function by inhibiting the TLR4/NF-κB/NLRP3/pyroptosis signaling pathway in the liver. This Syn is as effective as prednisone in treating AIH without side effects. Based on these findings, this novel Syn represents a potential therapeutic agent for AIH in clinical practice.

## INTRODUCTION

Autoimmune hepatitis (AIH) is a chronic inflammatory liver disease characterized by liver immune tolerance failure, leading to the destruction of the hepatic parenchyma (1, 2). Although corticosteroids are a common treatment for AIH, some patients react adversely to this treatment, and some patients may face severe adverse effects or recurrence after discontinuing steroid use (2–4). Thus, novel therapies are needed.

The precise etiology of AIH is unknown; however, research conducted over the past 4 decades has revealed that the interaction between genetic and environmental factors is central to its pathogenesis. The pathogenesis may be related to many mechanisms, such as immune imbalance, intestinal mucosal barrier destruction, and bacterial ecological imbalance (1, 5). Accumulating evidence shows that intestinal microflora is an important environmental factor influencing AIH. An increase in harmful intestinal bacteria can activate inflammatory pathways and disrupt the intestinal barrier, leading to intestinal bacterial transfer, the introduction of bacterial lipopolysaccharide into blood circulation, and an increase in intestinal permeability (6–8). Therefore, much research has focused on various probiotics, prebiotics, and synbiotics to restore gut microbiota (9, 10). These beneficial effects are attributed directly to an improved intestinal microbial environment from ingesting probiotics, prebiotics, and synbiotics (11–13).

**FIG 1:**
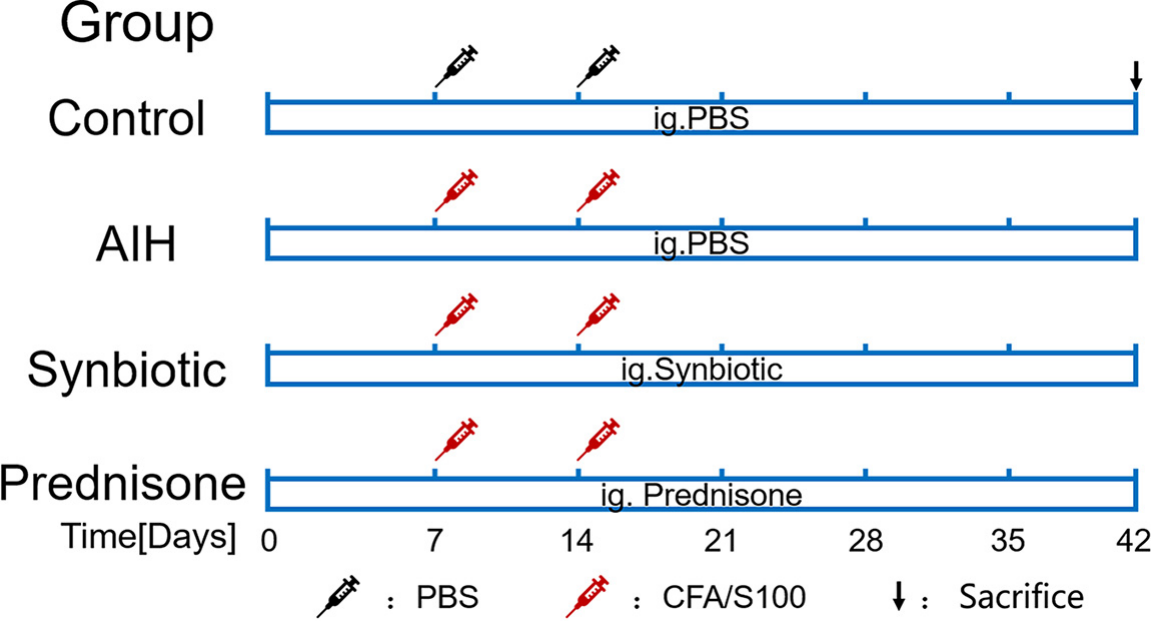
Schematic illustration of the experimental flow. S100/CFA or PBS were injected into mice on days 7 and 14. Mice were treated every day for 42 days (from day 1 to the end of the experiment) by oral gavage (see Materials and Methods for details). The mice were sacrificed on day 42, and the feces, blood, liver, ileum, colon, and spleen were harvested. ig, intragastrical administration; *n* = 10.

Synbiotics are a mixture of probiotics and prebiotics that can more fully regulate beneficial bacteria, leading to significant benefits (14). *Lactobacillus* and *Bifidobacterium* species are the most commonly used probiotics (15, 16). They colonize the intestinal tract and modulate gut microbiota (17). Recent studies revealed that the prebiotics konjac glucomannan oligosaccharides (KGMO) help regulate the gut microbiota and inflammation and enhance the mucosal barrier (18–20). The role of a novel synbiotic containing two probiotic strains (Lactobacillus acidophilus and Bifidobacterium infantis) and KGMO in alleviating AIH is unknown. Thus, we investigated the effects of the combination of L. acidophilus, *B. infantis*, and KGMO on the liver and the role of this synbiotic in regulating intestinal flora and maintaining intestinal integrity in the S100/complete Freund’s adjuvant (CFA)-induced AIH mouse model (Fig. 1).

## RESULTS

### Synbiotic treatment ameliorated liver injury and inflammation in AIH mice.

**FIG 2:**
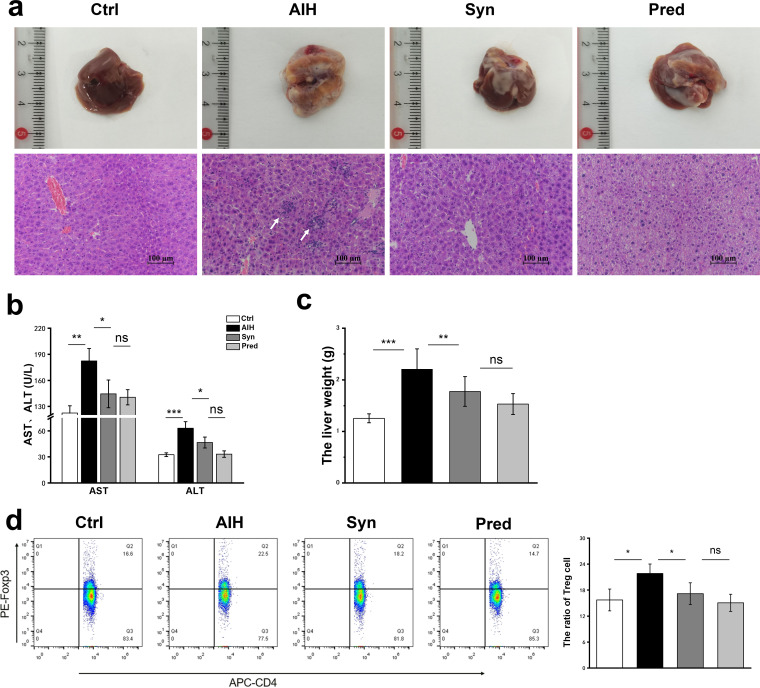
Synbiotics ameliorates liver injury in AIH mice. (a) Liver photographs and H&E-stained sections of livers. (b) The serum concentrations of ALT and AST. (c) Liver weight. (d) Representative flow cytometry plots and the percentage of Treg cells out of the spleen CD4^+^ population were calculated. Data are expressed as means ± SEM (*n* = 7). *, *P*< 0.05; **, *P* < 0.01; ***, *P* < 0.001; ns, not significant.

Mice were administered S100/CFA twice by intraperitoneal injection to generate the AIH model. There was no difference in the body weight or food intake between the control (Ctrl), AIH, synbiotic (Syn), and prednisone (Pred) groups (see Fig. S1a and b in the supplemental material). The livers of the AIH group mice were abnormally lobulated and paler in color than those of Ctrl mice, which had a bright red soft texture and smooth surface. The livers from mice in the Syn and Pred groups were similar to those of normal mice (Fig. 2a). Liver weight was significantly increased in the AIH group compared with that of the Ctrl group, and Syn treatment remarkably decreased liver weight (Fig. 2c). Hematoxylin and eosin (H&E) staining further revealed severe infiltration by inflammatory cells in the livers of mice in the AIH group (Fig. 2a). Treatment with Syn or Pred alleviated liver inflammation; however, prednisone caused significant edema. Serum assays were performed to assess hepatic dysfunction. As shown in Fig. 2b, Syn significantly decreased the serum alanine aminotransferase (ALT) and aspartate aminotransferase (AST) levels that AIH increased. These data indicated that the major histopathological and serological changes of AIH were generated in mice by S100/CFA treatment. These changes could be reversed by treatment with Syn.

10.1128/msystems.01127-22.1 FIG S1 (a) Changes in body weights. (b) Average food intake per group during the experiment. Data are expressed as means ± SEM (*n* = 7). *, *P* < 0.05; **, *P* < 0.01; ***, *P* < 0.001. Download FIG S1, TIF file, 0.2 MB. Copyright © 2023 Kang et al. 2023 Kang et al. https://creativecommons.org/licenses/by/4.0/ This content is distributed under the terms of the Creative Commons Attribution 4.0 International license.

Regulatory T (Treg) cells expressing transcription factor Foxp3 play a key role in limiting inflammatory responses in the intestine (21). Therefore, we measured the percentage of Treg cells in the spleen via flow cytometry and found that the percentage of Treg cells in the AIH group was elevated compared with that of the controls. Interestingly, Syn treatment counteracted this elevation (Fig. 2d).

**FIG 3:**
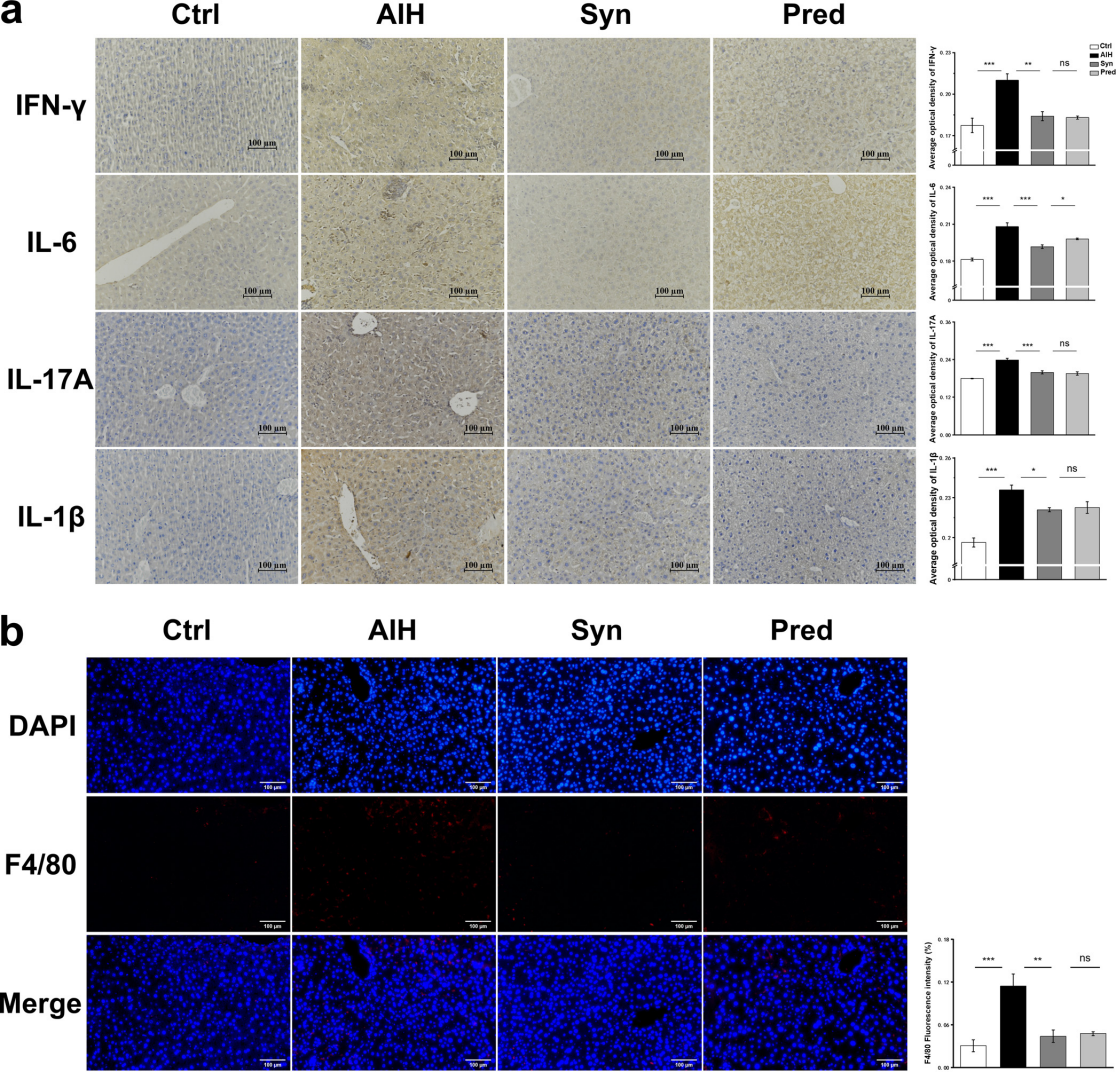
Synbiotics treatment relieves hepatic inflammation. (a) Immunohistochemistry for IFN-γ, IL-6, IL-17A, and IL-1β protein in hepatic tissue is shown. (b) Immunofluorescence for F4/80 protein in hepatic tissue is shown.

The inflammatory reaction is a major feature of liver injury from AIH. To determine whether Syn could inhibit the hepatic inflammatory responses in AIH, we measured the hepatic levels of inflammatory cytokines. The hepatic levels of all inflammatory cytokines (interferon gamma [IFN-γ], interleukin-6 [IL-6], IL-17A, and IL-1β) were higher in the AIH group than those in the Ctrl group (Fig. 3a). Syn reduced these levels in the liver. In addition, immunofluorescence staining of inflammatory cytokines in F4/80 cells indicated that the liver macrophages in the AIH group had a more severe inflammatory signature than those of the Ctrl group. In addition, Syn significantly decreased F4/80 expression in the hepatic tissues (Fig. 3b). These data suggest that Syn could reduce inflammation in AIH mice.

### Synbiotic treatment inhibited NLRP3 inflammasome activation and reduced pyroptosis in AIH mice.

**FIG 4:**
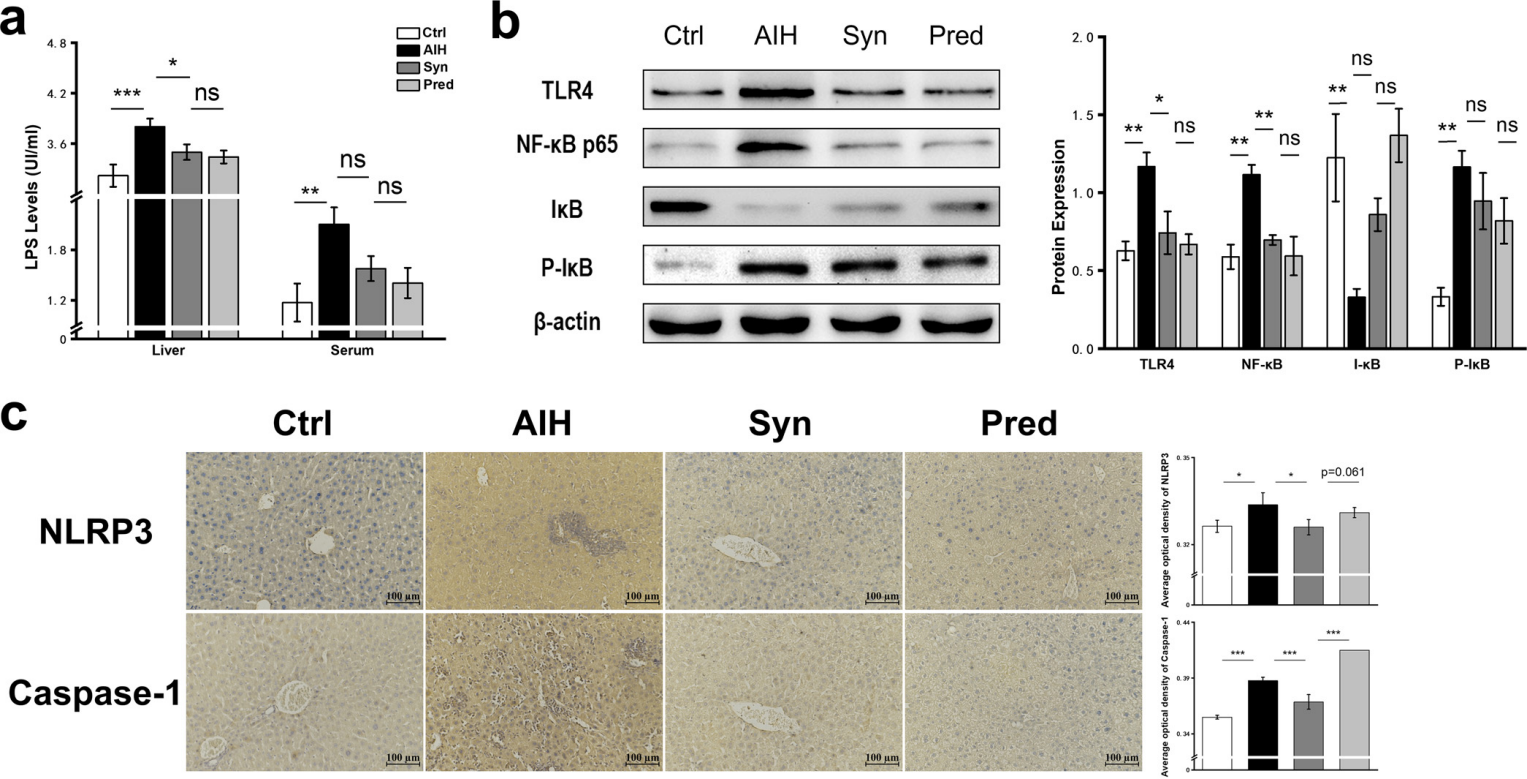
(a) LPS levels in serum and liver. (b) The protein expression of TLR4, NF-κB, IκB, and P-IκB was measured by Western blot. (c) Immunohistochemistry for NLRP3 and caspase-1 protein hepatic tissue.

LPS-induced TLR4 signaling has been observed in AIH (22). Our data demonstrated that liver tissue from AIH animals had not only a dramatic increase in the expression of TLR4/NF-κB signaling pathway components but also increased NLRP3 inflammasome activation and pyroptosis. LPS levels were increased in serum and liver homogenates of AIH mice; however, these levels decreased after treatment with Syn (Fig. 4a). TLR4, the receptor of LPS (23), was also activated in the AIH group (Fig. 4b). NF-κB is a downstream TLR4 biomarker, and IκB and P-IκB are downstream biomarkers of NF-κB; NF-κB can mediate the phosphorylation of IκB (24). Syn reduced NF-κB expression and P-IκB levels and increased IκB expression. Therefore, Syn reduced liver inflammation by inhibiting NF-κB activity in AIH mice.

NF-κB activation upregulates NLRP3 protein expression, promoting more IL-1β production and pyroptosis (25–27). Thus, we investigated whether Syn could inhibit the activation of the NLRP3 inflammasome. Immunohistochemistry showed that NLRP3, caspase-1, and IL-1β expression levels were significantly increased in the AIH group compared with those in the Ctrl group (Fig. 4c and Fig. 3a). These increases were reduced by Syn treatment, suggesting that Syn reduced liver damage in the AIH mouse model by downregulating the expression of the NLRP3 inflammasome, signaling pathway components to inhibit pyroptosis.

### Synbiotic treatment reversed gut microbiota dysbiosis in AIH mice.

**FIG 5:**
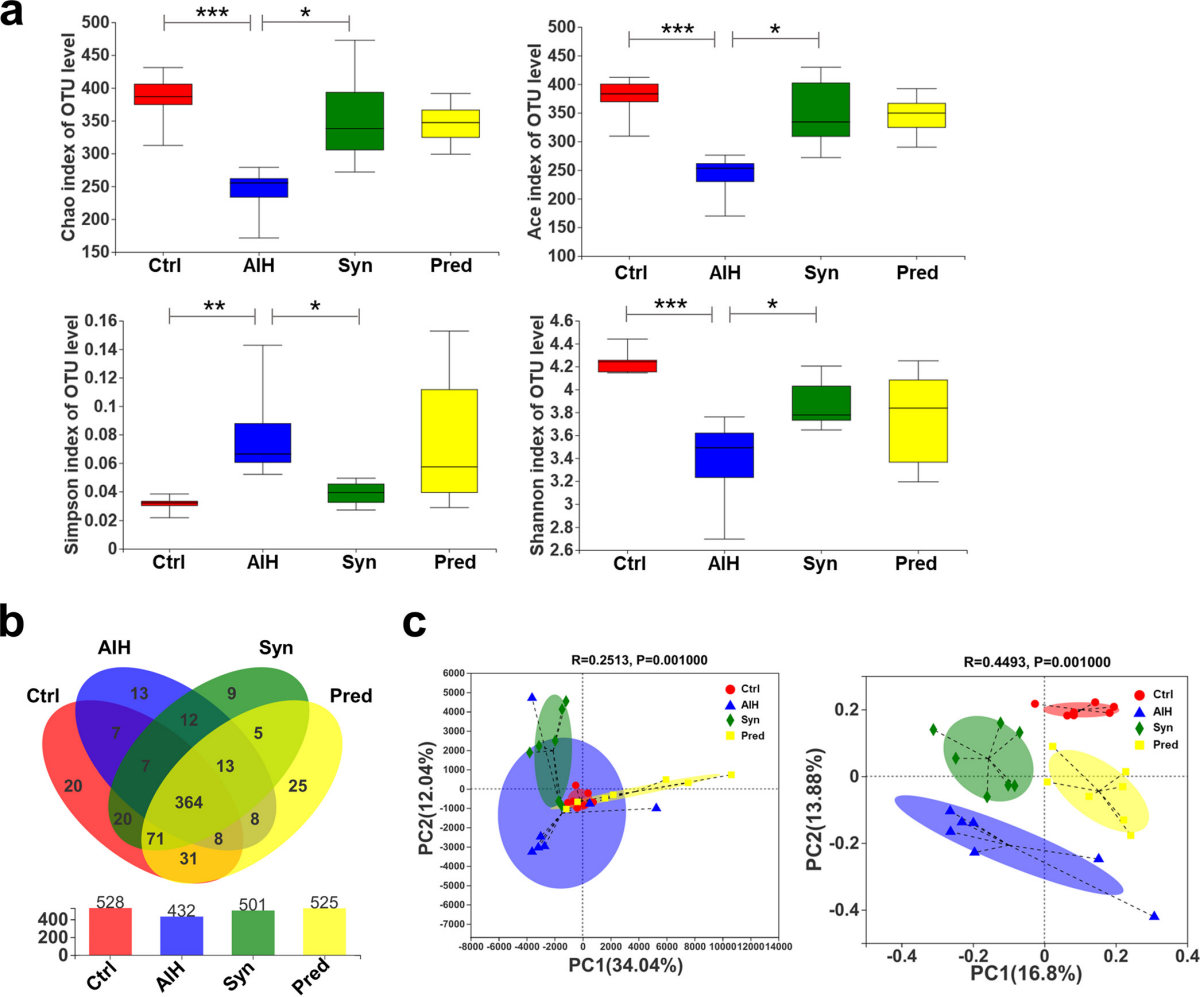
(a) The gut microbiomes were analyzed for α-diversity measures, including Chao, ACE, and Simpson and Shannon indices. Data are expressed as means ± SEM (*n* = 7). *, *P*< 0.05; **, *P* < 0.01; ***, *P* < 0.001. (b) Venn diagram illustrates the overlap of OTUs in intestinal microbiota among the samples. (c) PCA, principal-component analysis (left); PCoA, principal coordination analysis (right).

**(i) Synbiotic treatment changed the diversity and richness of the gut microbiota in AIH mice.** The α-diversity metrics (operational taxonomic units [OTUs], Chao1, and the abundance-based coverage estimator [ACE]) of the AIH group were significantly lower than those of the Ctrl group, as was evenness based on the Shannon and Simpson indices, whereas Syn treatment completely reversed these effects (Fig. 5a). Based on the OTU abundance information, we performed Venn diagram analysis to compare the common or unique OTU characteristics between the four groups. All four groups had specific microbial community characteristics (Fig. 5b). A total of 613 OTUs were identified from all fecal samples; 364 OTUs existing in all groups were defined as core OTUs. The core OTUs comprised approximately 59% of the total OTUs. These OTUs were markedly increased after Syn treatment.

Principal-component analysis (PCA) and principal coordinates analysis (PCoA) were performed to further analyze the differences between groups (Fig. 5c). The distance between the dots with different colors showed the similarity of their own bacterial community structure. Dots tended to gather together within their respective groups. Collectively, these data indicated that Syn markedly improved the richness and diversity of the intestinal microbiota.

**FIG 6:**
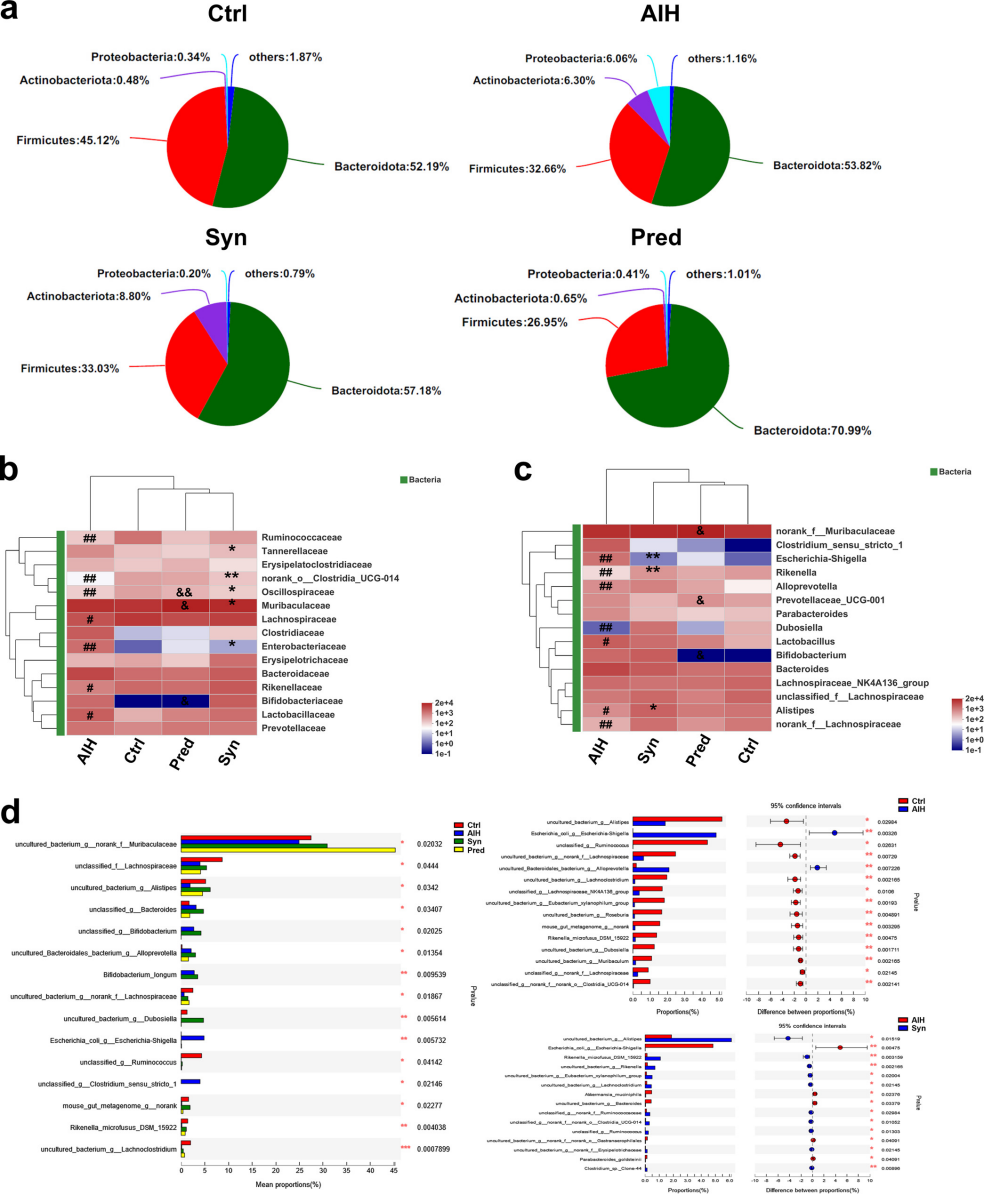
Synbiotics alters gut microbiota composition in AIH mice. Effects of synbiotics treatment on the average relative abundance of the major microbial phyla (a), relative abundance of the top 15 different families (b), relative abundance of the top 15 different genera (c), and relative abundance of the top 15 different species (d). Data are expressed as means ± SEM (*n* = 7). *, *P* < 0.05; **, *P* < 0.01; ***, *P* < 0.001. *, AIH-Syn; &, Syn-Pred; #, AIH-Ctrl.

**(ii) Synbiotic treatment altered the composition of the gut microbiota in AIH mice.** According to previous studies, the depletion of beneficial bacteria and expansion of potential pathobionts are associated with AIH disease status (7, 28). Thus, we analyzed the gut microbiota composition in the groups at the phylum, family, genus, and species levels. The microbiomes of all groups were predominantly the phyla *Bacteroidetes*, *Firmicutes*, *Actinobacteriota*, and *Proteobacteria* (Fig. 6a). We also compared three bacteria (*Proteobacteria*, *Patescibacteria*, and *Deferribacterota*) that were present at lower levels (see Fig. S2a to c in the supplemental material). *Proteobacteria* are Gram-negative bacteria composed mainly of lipopolysaccharides that include pathogenic species exhibiting proinflammatory effects, such as Escherichia coli (29). Notably, there was a higher abundance of *Proteobacteria* and a lower abundance of *Patescibacteria* and *Deferribacterota* in the AIH group than those in the Ctrl group; however, normal levels of these three bacteria were restored completely by Syn treatment.

10.1128/msystems.01127-22.2 FIG S2 The average relative abundance of the *Proteobacteria*, *Patescibacteria*, and *Deferribacterota*. *, *P* < 0.05; **, *P* < 0.01; ***, *P* < 0.001. Download FIG S2, TIF file, 0.1 MB. Copyright © 2023 Kang et al. 2023 Kang et al. https://creativecommons.org/licenses/by/4.0/ This content is distributed under the terms of the Creative Commons Attribution 4.0 International license.

In addition to the phyla, the families, genera, and species were evaluated (Fig. 6b to d). Of the top 15 families, the AIH group had decreased relative abundances of *Ruminococcaceae* and *norank_o_Clostridia_UCG-014* compared with the Ctrl group, whereas *Lactobacillaceae* and *Enterobacteriaceae* were markedly increased. Syn treatment restored *norank_o_Clostridia_UCG-014*, *Oscillospiraceae*, and *Enterobacteriaceae* levels. Of the top 15 genera, the relative abundances of *Rikenella*, *Dubosiella*, *Alistipes*, and *norank_f_Lachnospiraceae* were decreased in the AIH group compared with those in the Ctrl group, whereas Escherichia*-Shigella*, *Alloprevotella*, and *Lactobacillus* were increased. Syn restored Escherichia*-Shigella*, *Rikenella*, and *Alistipes* to normal levels. The relative abundance of all 15 species in the AIH group was significantly different than that in the Ctrl group. Two species (Escherichia*_coli_g_Escherichis-Shigella* and *uncultured_Bacteroidales_bacterium_g_Alloprevotella*) were significantly increased, whereas the others were drastically decreased. Syn treatment of AIH mice noticeably increased 10 species and decreased others considerably. There were also slight differences between the Syn and Pred groups (see Fig. S3 in the supplemental material). We built a bacterial abundance correlation network to study the interactions between these bacterial species. Network analysis revealed that the significant roles of these interactions were *unclassified_f__Lachnospiraceae*, *unclassified_g_Bacteroides*, *uncultured_bacterium_g_Lachnospiraceae_NK4A136_group*, and *uncultured_bacterium_g_Alistipes* (see Fig. S5 in the supplemental material).

10.1128/msystems.01127-22.3 FIG S3 Relative abundance of the top 15 different species between the Syn and Pre groups. Download FIG S3, TIF file, 0.5 MB. Copyright © 2023 Kang et al. 2023 Kang et al. https://creativecommons.org/licenses/by/4.0/ This content is distributed under the terms of the Creative Commons Attribution 4.0 International license.

10.1128/msystems.01127-22.5 FIG S5 Effect of synbiotics on the Gram-negative and pathogenic potential of BugBase-based gut microbiota between the Ctrl and AIH groups. Download FIG S5, TIF file, 0.4 MB. Copyright © 2023 Kang et al. 2023 Kang et al. https://creativecommons.org/licenses/by/4.0/ This content is distributed under the terms of the Creative Commons Attribution 4.0 International license.

**FIG 7:**
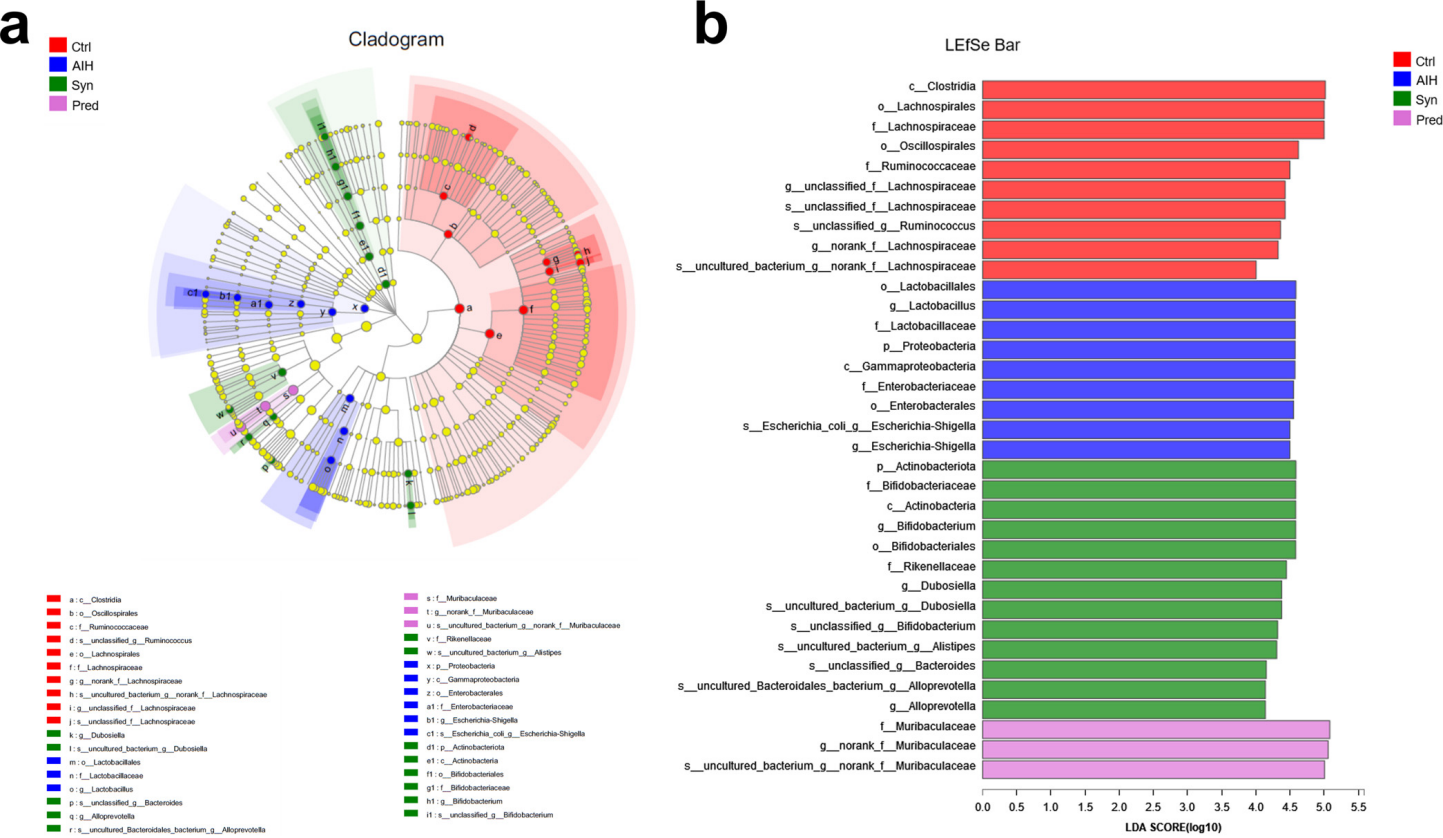
Synbiotics alterations on gut microbiota biomarkers in AIH mice. Identification was conducted of discriminant taxa among the four groups by LDA effect size (LEfSe) analysis. (a) Cladogram of the microbiota. Significant discriminant taxon nodes of the Ctrl, AIH, Syn, and Pred are represented by red, blue, green, and pink, respectively. Nondiscriminant taxon nodes are represented by yellow. Branch areas are shaded according to the highest ranked variety for that taxon. (b) The LDA score indicates the level of differentiation among the four groups. A threshold value of 4.0 was used as the cutoff level. The horizontal bar chart shows discriminant taxa. Significant discriminant taxa of the Ctrl, AIH, Syn, and Pred groups are represented by red, blue, green, and pink, respectively.

To obtain further information on the effects of the different groups on each taxon, linear discriminant analysis effect size (LEfSe) was performed, and linear discriminant analysis (LDA) scores were generated (Fig. 7a and b). The LEfSe method is a combination of a nonparametric test and LDA. It is suitable for detecting differences in bacterial abundance. An LDA value greater than 4.0 was used as the screening standard to determine the abundance of microorganisms in a group. Taxa had differential abundance in the four groups. *Clostridia* and *Oscillospirales* had the highest significance in the Ctrl group, whereas *Lactobacillales* and *Lactobacillus* represented the major populations in the AIH group. The major populations of the Syn group consisted of *Actinobacteriota*, *Bifidobacteriaceae*, and *Bifidobacterium*. *Muribaculaceae* were vital and could serve as a biomarker in the Pred group. Furthermore, the number of taxa with differential abundances in the AIH group was higher than that in the Syn group, which shows that Syn has a recovery effect on the increases of some microbiota caused by AIH. Together, these data showed that Syn could restore intestinal flora composition.

**(iii) Synbiotic treatment altered gut microbiota function and phenotype in AIH mice.** The intestinal microbiota contributes to the onset and progression of AIH. Numerous studies support the intimate linkage between the etiology of AIH and intestinal microbiota. Therefore, PICRUSt was employed to predict the functional potential of bacteria in the AIH group. Further analysis was performed using the Kyoto Encyclopedia of Genes and Genomes (KEGG) database, and the enzyme, KEGG orthology (KO), modules, and pathways belonging to the KEGG functional categories were identified. The AIH group had four functional categories distinct from the Ctrl group. Syn treatment largely abolished these differences (Fig. S4a to d). Thus, Syn treatment improves gut microbiota functions involving inflammatory injury, metabolism, immune response, and pathopoiesia.

10.1128/msystems.01127-22.4 FIG S4 Synbiotics effects on gut microbiota function basing Kyoto Encyclopedia of Genes and Genomes (KEGG) database in AIH mice. Effects of synbiotics treatment on enzyme (a); KEGG orthology (KO) (b), module (c), and pathways 1, 2, and 3 (d). *, *P* < 0.05; **, *P* < 0.01; ***, *P* < 0.001. Download FIG S4, TIF file, 5.0 MB. Copyright © 2023 Kang et al. 2023 Kang et al. https://creativecommons.org/licenses/by/4.0/ This content is distributed under the terms of the Creative Commons Attribution 4.0 International license.

**FIG 8:**
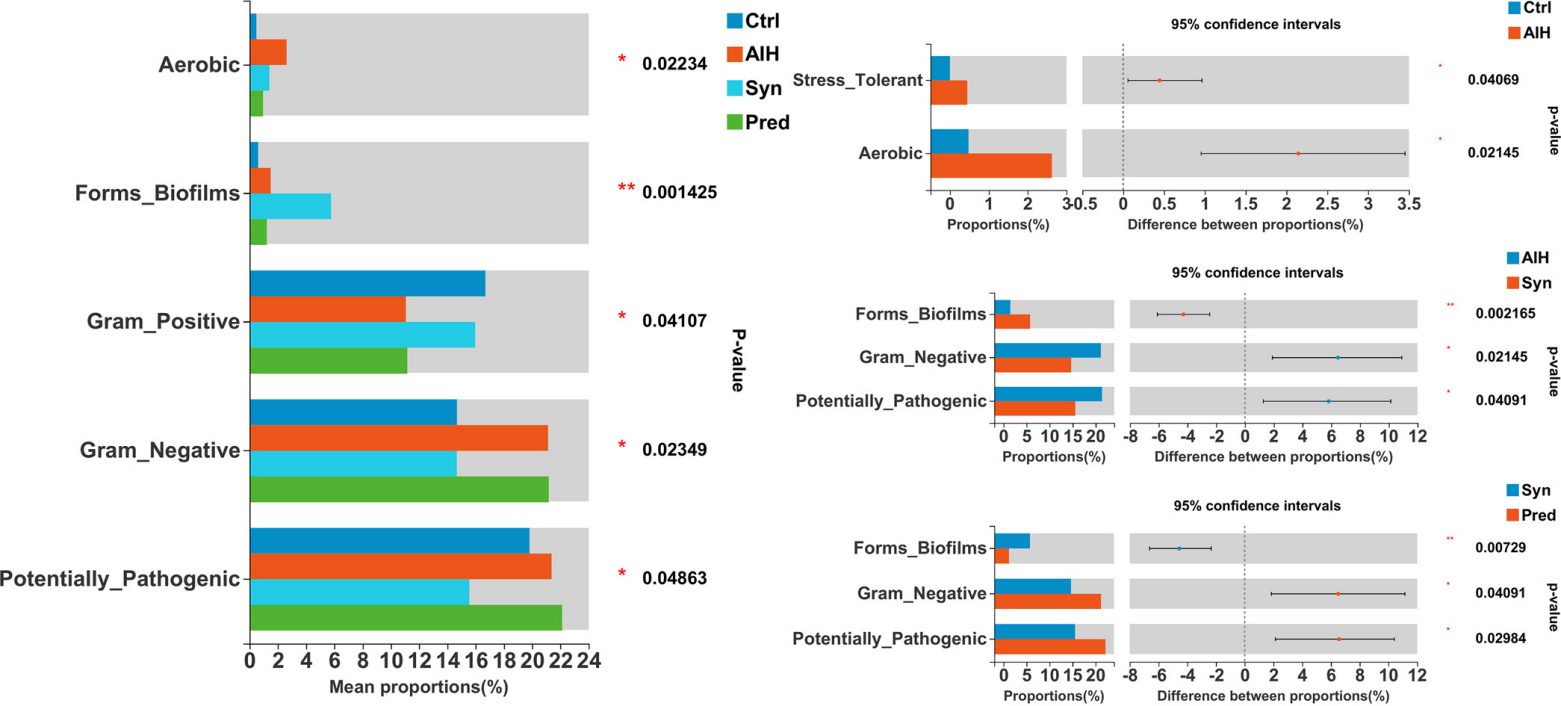
Synbiotics effects on gut microbiota phenotype based on BugBase, including aerobic, Gram-positive, Gram-negative, biofilm-forming, and pathogenic potential. *, *P* < 0.05; **, *P* < 0.01; ***, *P* < 0.001.

We evaluated the presence of aerobic, biofilm-forming, Gram-negative, Gram-positive, and potentially pathogenic bacteria to predict the organism-level coverage of functional pathways and biointerpretable phenotypes (Fig. 8). We observed that the proportion of stress-tolerant and aerobic bacterium types significantly increased in the AIH group compared with that in the Ctrl group. Moreover, Gram-negative (*P* = 0.07364) and potentially pathogenic bacteria (*P* = 0.3711) increased modestly in the AIH group, but the changes were not statistically significant compared with the Ctrl group (see Fig. S6 in the supplemental material). Interestingly, Syn treatment significantly decreased the Gram-negative and potentially pathogenic bacteria.

10.1128/msystems.01127-22.6 FIG S6 Network of bacteria. Analyzed by Spearman rank correlation analysis. The size of the circle represents the average abundance of the species; the line represents the correlation between the two species; the thickness of the line represents the strength of the correlation; and regarding the color of the line, red represents a positive correlation, and green represents a negative correlation. Download FIG S6, TIF file, 0.4 MB. Copyright © 2023 Kang et al. 2023 Kang et al. https://creativecommons.org/licenses/by/4.0/ This content is distributed under the terms of the Creative Commons Attribution 4.0 International license.

### Synbiotic treatment improved intestinal damage and gut barrier function in AIH mice.

In addition to remodeling the microbiota composition and function of the gut, synbiotics can also enhance intestinal barrier functions, which might be essential for preventing the entry of harmful intestinal content into the bloodstream. Thus, we explored whether the new synbiotic could lessen autoimmune liver injury by strengthening the intestinal barrier function.

**FIG 9:**
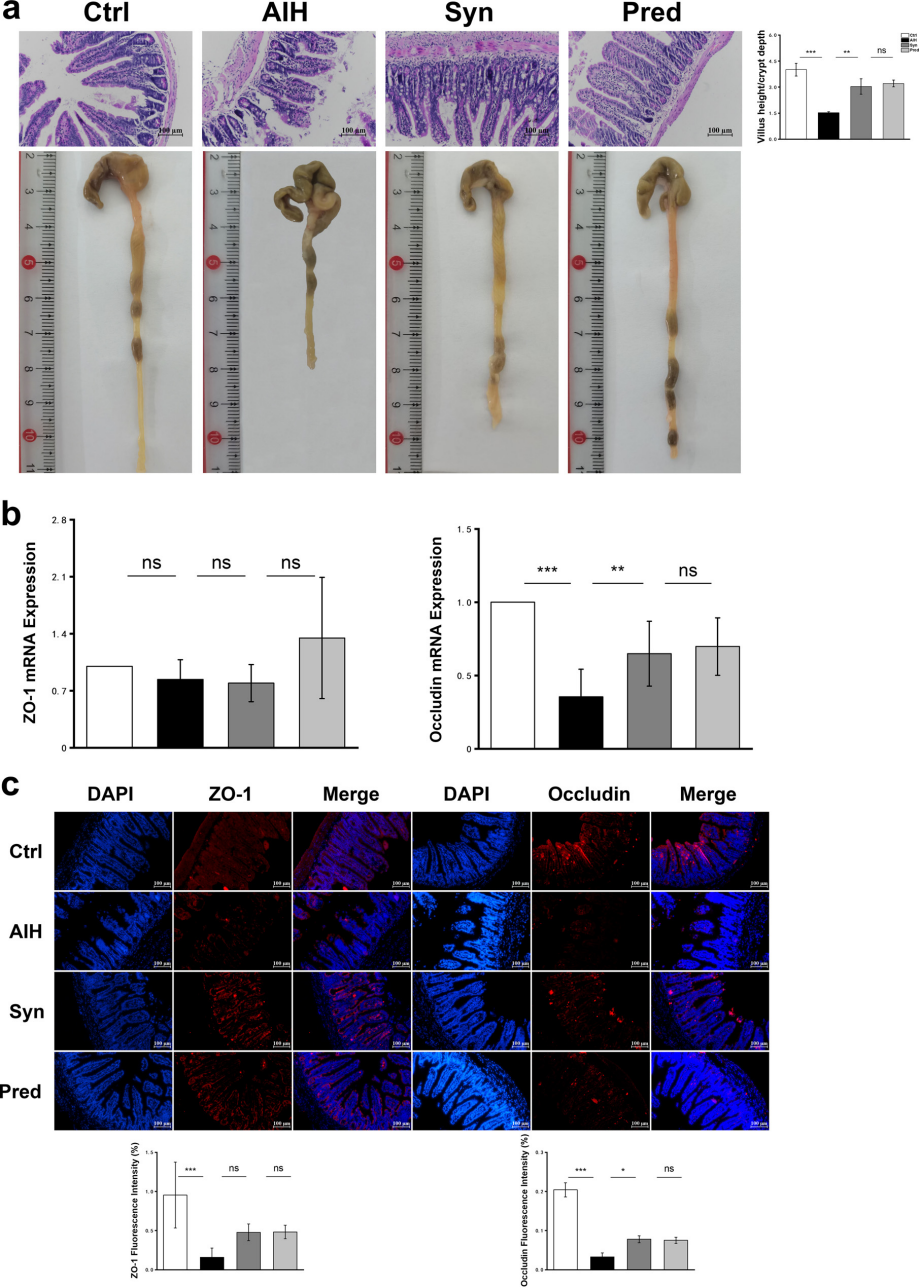
Synbiotics restore intestinal barrier function in AIH mice. Effects of synbiotics treatment on the representative images of ileum tissue H&E staining, colonic length, and the ratio of villus length to crypt depth (a); relative expression of gene Occludin and ZO-1 in ileum (b); and immunofluorescence expression of ZO-1 and Occludin in ileum (c). Data are expressed as means ± SEM (*n* = 7). *, *P* < 0.05; **, *P* < 0.01; ***, *P* < 0.001.

Histopathology of the intestinal mucosa in the Ctrl group showed that the intestinal layer had a clear structure with intact surface epithelium, neatly arranged intestinal villi, and no destruction of the intestinal wall structure. In contrast, the structure of the intestinal wall in the AIH group was damaged, the intestinal villi were fractured, and the colon length was significantly reduced; Syn treatment completely restored the intestinal mucosa (Fig. 9a). However, H&E staining of the colon tissue showed no obvious morphological differences between the four groups (see Fig. S7 in the supplemental material). Thus, we focused on changes in the ileum. We calculated the ratio of villus height to crypt depth to evaluate intestinal morphological alterations. This ratio in the AIH group was significantly lower than that of the Ctrl group, whereas Syn treatment of AIH mice restored the ratio to close to that of the Ctrl group (Fig. 9a). Because endothelial barrier integrity depends on the interaction between occludin and zonula occludens-1 (ZO-1), we evaluated ZO-1 and occludin in the four groups (Fig. 9b and c). Immunofluorescence showed that ZO-1 and occludin decreased in the AIH group compared with those in the Ctrl group but increased in the Syn group compared with those in the AIH group. The same results were obtained by quantitative real-time PCR. These data indicated that the synbiotic could repair the intestinal barrier dysfunction of AIH mice.

10.1128/msystems.01127-22.7 FIG S7 Representative images of colon tissue H&E staining. Download FIG S7, TIF file, 1.6 MB. Copyright © 2023 Kang et al. 2023 Kang et al. https://creativecommons.org/licenses/by/4.0/ This content is distributed under the terms of the Creative Commons Attribution 4.0 International license.

### Correlation analysis of gut microbiota, liver weight, blood parameters, bacterial translocation, and protein and gene expression.

**FIG 10:**
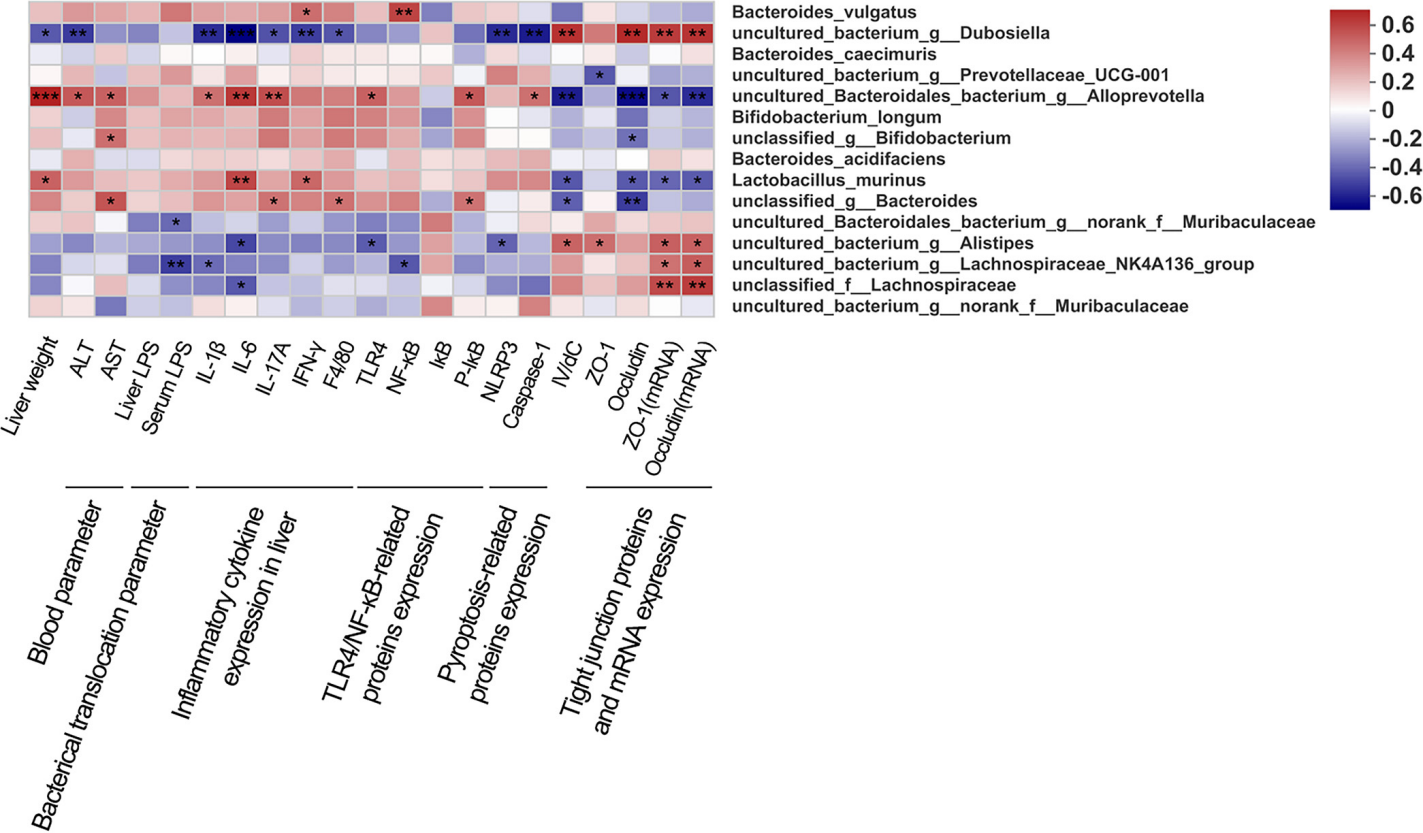
The relationship between liver weight, blood parameters, degree of bacterial translocation, inflammatory response, TLR4/NF-κB/NLRP3 pathway and ileum barrier function, and the 15 top species is estimated by Spearman’s correlation analysis. *, *P* < 0.05; **, *P* < 0.01; ***, *P* < 0.001. lV/dC, the ratio of villus length to crypt depth.

The correlations between intestinal microbiota and liver weight, blood parameters, degree of bacterial translocation, and protein and gene expression were investigated based on the heatmap (Fig. 10). Among the 15 highly abundant species, 11 species were notably associated with mouse-related parameters. Bacteroides vulgatus, *uncultured_Bacteroidales_bacterium_g_Alloprevotella*, *unclassified_g_Bifidobacterium*, Lactobacillus murinus, *unclassified_g_Bacteroides*, and *uncultured_bacterium_g_Prevotellaceae_UCG-001* were significantly positively correlated with liver weight, blood parameters, degree of bacterial translocation, inflammatory cytokine expression in liver, and TLR4/NF-κB- and pyroptosis-related protein expression but negatively correlated with lV/dC and ileum tight junction protein and mRNA expression. Thus, these bacterial species might induce AIH. In contrast, *uncultured_bacterium_g_Dubosiella*, *uncultured_Bacteroidales_bacterium_g_norank_f_Muribaculaceae*, *uncultured_bacterium_g_Alistipes*, *uncultured_bacterium_g_Lachnospiraceae_NK4A136_group*, and *unclassified_f_Lachnospiraceae* had the opposite positive and negative correlations and, thus, might alleviate AIH. Taken together, these findings demonstrated that 11 bacterial species play a crucial role in AIH development.

## DISCUSSION

In recent years, research on the relationship between the gut microbiome and AIH has accumulated rapidly, accompanied by increasing interest in treating AIH by modulating the gut microbiota. A synbiotic is a mixture of one or more probiotics and prebiotics that beneficially affect the host by promoting the survival and colonization of live microbes in the gastrointestinal tract (30). Therefore, synbiotics may be the most effective way to treat AIH by regulating the gut microbiota without side effects.

Because of gut-liver cross-talk and the decreased abundance of probiotics, especially *Bifidobacterium* and *Lactobacillus*, in the feces of AIH patients, we speculated that probiotic supplementation could improve intestinal microecology, enhance intestinal wall structure and function, and alleviate AIH. Our results demonstrated that a synbiotic containing L. acidophilus and *B. infantis* could alleviate AIH, confirming our hypothesis. In the present study, oral administration of the new synbiotic alleviated liver injury and improved liver function concomitant with a reduction in hepatic inflammation and pyroptosis in an S100/CFA-induced AIH mouse model. This synbiotic also regulated the composition of the gut microbiota and maintained the integrity of the intestinal barrier.

Specifically, Syn treatment significantly decreased liver morphological changes, serum ALT and AST levels, and liver weight (Fig. 2a to c). Furthermore, this treatment markedly downregulated proinflammatory factors and macrophage infiltration marker protein F4/80 expression in hepatic tissue (Fig. 3a and b). Recently, some studies have shown that liver inflammation in AIH mouse models is associated with dysbiosis of the gut microbiota and increased LPS levels in the blood (7, 31, 32). LPS is a component of the Gram-negative bacterial cell wall and an endotoxin; its release results from the lysis of Gram-negative bacteria (33, 34). The gut is the source of most plasma LPS because the gut microbiota is the largest reservoir of Gram-negative bacteria in the body (35, 36). LPS is rarely in serum under normal circumstances. When the intestinal barrier integrity is damaged, serum LPS levels increase. Interestingly, we found that Syn treatment improved the integrity of the intestinal barrier and reduced serum LPS, TLR4/NF-κB signaling pathway activity, and inflammation in the AIH mouse model. Furthermore, our results indicated that Syn supplementation enhanced beneficial bacteria (e.g., *Alistipes and Rikenella*) and reduced potentially harmful bacteria (e.g., Escherichia*-Shigella* and Gram-negative bacteria) and inflammation in AIH mice. Therefore, the beneficial effects of Syn could be attributed to specific changes in the gut microbiota and the maintenance of intestinal barrier integrity.

The TLR4/NF-κB pathway is a major signal transduction pathway in AIH (22). The present study demonstrated that Syn treatment could suppress TLR4/NF-κB signaling in AIH. Moreover, the NLRP3 inflammasome can activate caspase-1, leading to the maturation of IL-1β and the induction of the pyroptosis response through the NF-κB signaling pathway (37), which is consistent with our results (Fig. 4a to c).

Treg cells are immune suppressor cells that play an important role in maintaining immune homeostasis and tolerance in the liver (55, 56). In patients with hepatitis B-associated hepatocellular carcinoma, increased Treg cell prevalence is associated with CD8^+^ T-cell impairment and poor survival (38). Treg cells can also dampen ischemic injury in other organs, including the liver (39). Liu et al. (22) found that the proportion of Treg cells decreased in S100/CFA-induced AIH mice compared with that in normal mice. However, we obtained the opposite results in the present experiment (Fig. 2d). According to previous studies, Treg cells have an activated phenotype due to the greater tension imposed on regulatory mechanisms in chronic inflammation (40). Thus, it is perhaps unsurprising that the proportion of Treg cells increased in the AIH mice.

Recently, some studies have shown that the gut microbiota plays an important role in the occurrence and development of AIH (7, 41). The gut microbiota is crucial for regulating inflammation and maintaining intestinal barrier function (12, 42, 43), which was supported by our study. Specifically, we observed that Syn treatment increased the diversity and richness of the gut flora of AIH mice (Fig. 5a to c). Previous studies showed that the abundance of *Dubosiella* and *Alistipes* positively correlates with inflammation (44, 45). At the same time, *Bifidobacterium* can reduce AIH (46). Consistent with these reports, we observed that Syn treatment markedly decreased the abundance of *Dubosiella* and *Alistipes* and increased *Bifidobacterium* in AIH mice models (Fig. 6c). It has been reported that the number of *Lactobacillus* bacteria positively correlates with the expression of tight junction proteins in the intestine (47). Interestingly, we found that *Lactobacillus* was negatively correlated with the mRNA expression of tight junction proteins in the AIH mice model (Fig. 10).

Bacteria belonging to the *Lachnospiraceae* family are abundant members of the microbiota in healthy humans (48). *Lachnospiraceae* produce the short-chain fatty acids acetate and butyrate; butyrate reduces intestinal inflammation and bacterial translocation and facilitates colonization resistance against intestinal pathogens (49–51). Consistent with these observations, Syn supplement restored the percentage of *Lachnospiraceae* bacteria in the gut microbiota of AIH mice to levels close to that of normal mice (Fig. 6d). In addition, microbiome phenotype and bacterial functional potential prediction showed that Syn treatment of AIH mice reverted the phenotypes related to aerobic, potentially pathogenic, Gram-negative, and Gram-positive bacteria and functions involving inflammatory injury, inflammatory response, and pathopoiesia back to those observed in the Ctrl group (Fig. 8).

According to our results, Syn treatment could ameliorate liver injury and improve liver function in AIH mice. These beneficial effects of Syn may be relevant to restoring intestinal flora imbalance, improving intestinal barrier function, and alleviating liver inflammation. Above all, our findings showed that there was no significant difference between this new Syn and prednisone. However, histopathological analysis of mouse liver sections showed edema after prednisone treatment. Prednisone causes additional side effects, such as cushingoid features, facies lunaris, dorsal hump, striae, weight gain, acne, and hirsutism (52). Thus, Syn could be recommended for ameliorating AIH without these side effects.

**FIG 11:**
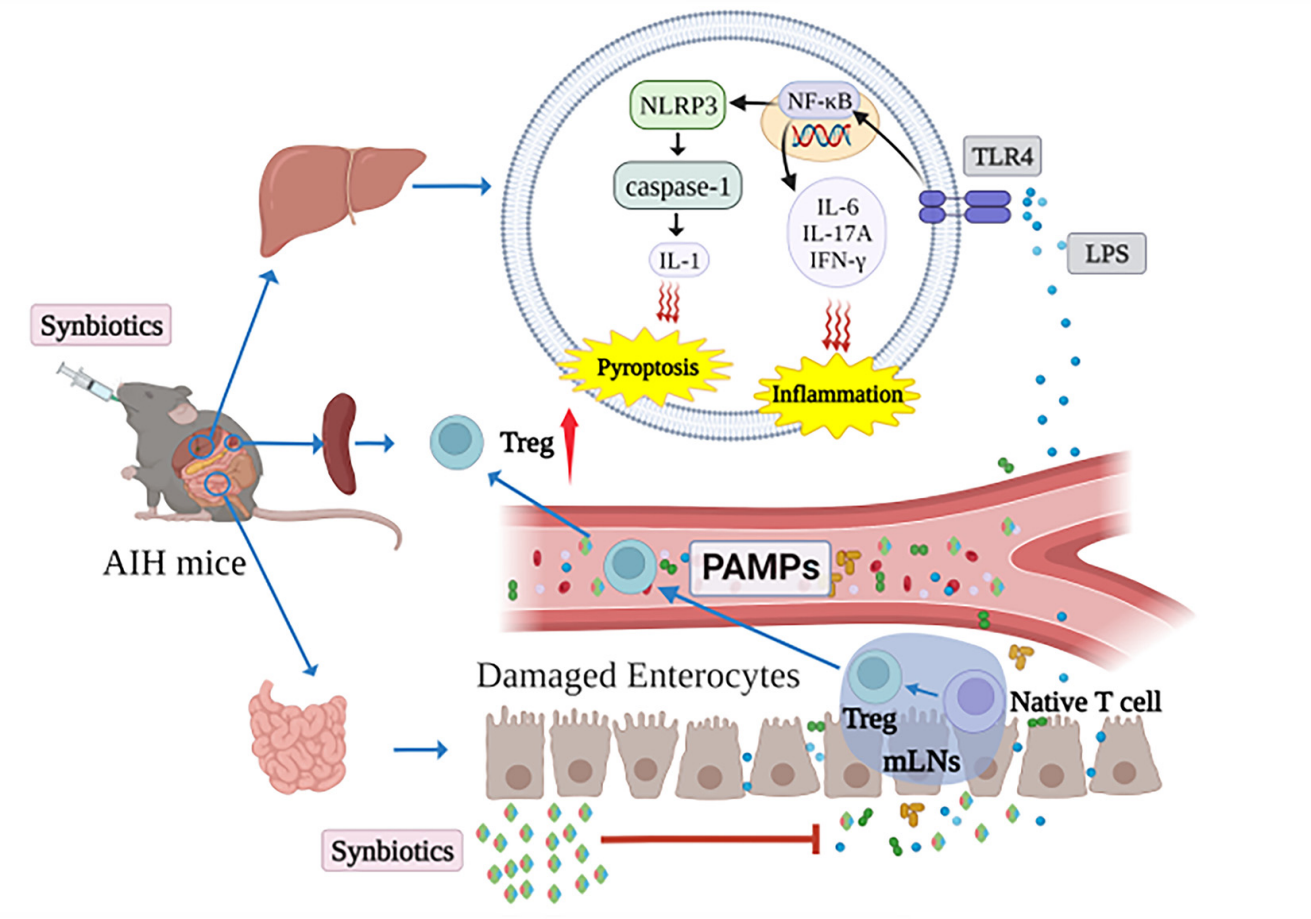
Mechanisms of protection of synbiotics against AIH mice. Synbiotics restore intestinal flora and intestinal barrier to inhibit bacteria and LPS to the liver. In addition, the TLR4/NF-κB/NLRP3 pyroptosis signaling pathway is suppressed via the synbiotics in the liver. Therefore, the synbiotics has an inhibitory effect on liver inflammation.

In summary, we found that Syn treatment could alleviate liver injury and improve liver function accompanied by reduced hepatic inflammation and pyroptosis. Our data indicated that Syn not only reversed gut dysbiosis but also maintained intestinal barrier integrity. Thus, its mechanism might be associated with modulating gut microbiota composition and intestinal barrier function by inhibiting the TLR4/NF-κB/NLRP3 pyroptosis signaling pathway in the liver (Fig. 11). Importantly, the new Syn presented in this study was as effective in treating AIH as prednisone but without the side effects. Therefore, this novel Syn represents a potential therapeutic agent for AIH in clinical practice.

## MATERIALS AND METHODS

### Preparation and administration of synbiotics.

The new synbiotics was prepared by us in the Department of Microbiology and Immunology, School of Basic Medical Sciences, Shanxi Medical University (China, Shanxi). The probiotic L. acidophilus (GenBank accession number OL457299) and *B. infantis* (GenBank accession number M58738) were isolated and stored at the Department of Microbiology and Immunology, School of Basic Medical Sciences, Shanxi Medical University (China, Shanxi). Then, L. acidophilus and *B. infantis* were grown in De Man, Rogosa and Sharpe (MRS) medium and Bifidobacteria medium with lactulose (BML), respectively, for a period of 48 h at 37°C under anaerobic conditions using the Anaerogen system (Oxoid, Basingstoke, UK). After that step, cultured cells were harvested by centrifugation (4,000 × *g* at 4°C for 5 min), washed three times with sterile phosphate-buffered saline (PBS), and then resuspended in sterile PBS. Konjac glucomannan oligosaccharide (KGMO) is a β-1,4 linked polysaccharide composed of a d-glucose (G) and d-mannoses (M) backbone lightly branched, with branches through β-1, 6-glucosyl units. The prebiotic KGMO was purchased from Lijiang Daran Biological Technology Corporation (Lijiang, China). For synbiotics treatment, it was suspended using a sterile PBS solution, including 1.5 × 10^9^ CFU L. acidophilus, 1.5 × 10^9^ CFU *B. infantis*, and 2 g/kg of body weight (the average weight of each group of mice) KGMO for each mouse per day.

### Animals.

Forty-three male specific-pathogen-free (SPF) C57BL/6 mice (4 weeks of age;18 to 20 g) were purchased from Laboratory Animal Center of Shanxi Medical University (Shanxi, China). Mice were given water and food under a controllable environment (temperature, 22 to ~25°C; humidity, 45% to ~55%; 12:12-h light-dark cycle). Before commencement of the AIH modeling, all mice were allowed to acclimatize to their new environment for 1 week. Then, 40 mice were divided randomly into four groups (*n* = 10 per group), as follows: control (Ctrl), synbiotics (Syn) treatment group, and prednisone (Pred) treatment. The remaining three mice were used to prepare a liver-specific antigen. The study was permitted by the ethical committee of Shanxi Medical University (permit number SYDL2021001).

### Experimental design and treatment.

To prepare the liver-specific antigen S100, three mice were selected randomly and sacrificed. The livers were perfused with phosphate-buffered saline (PBS) and removed as described previously and used to prepare fresh S100 antigen (22). In brief, the liver was cut into small pieces, homogenized, and centrifuged at 150 × *g* for 10 min to remove the tissue fragments. The supernatant was subsequently centrifuged for 1 h at 100,000 × *g* (Eppendorf, Germany). The supernatant was called S-100. The protein fraction was used at a concentration of 0.5 to 2.0 g/L. For the establishment of the AIH model, except for the Ctrl group, all mice were injected with a dose of 1 mL antigen (0.5 mL of syngeneic S-100 antigen was emulsified in an equal volume 0.5 mL of complete Freund’s adjuvant [CFA] on day 7 and day 14). The control group was injected intraperitoneally with 1 mL PBS. Mice of the Ctrl group and AIH group were gavaged with PBS (0.2 mL) every day from day 1 to the end of the experiment, the Syn group was gavaged with synbiotics (0.2 mL) that dissolved in PBS, and the Pred group was gavaged with prednisone (0.15 mg/kg) that dissolved in normal saline every day from day 1 to the end of the experiment. On the day 42, all the mice were sacrificed under anesthesia (Fig. 1). Blood samples were collected and centrifuged at 3,000 rpm at 4°C for 15 min to obtain the serum. Liver tissues and ileum tissues were either fixed in 4% paraformaldehyde for immunohistochemical and histological examination or frozen at –80°C.

### Serum and liver biochemical parameters.

Serum levels of alanine aminotransferase (ALT) and aspartate aminotransferase (AST) were determined using alpha-ketoglutarate and l-alanine as the substrates by using an automated chemistry analyzer (BioMajesty, Japan) in the TaiYuan Hospital of Traditional Chinese Medicine.

LPS levels in the serum and liver homogenate were measured using an enzyme-linked immunosorbent assay (ELISA) kit (ABmart, China), according to the manufacturer’s instructions. Briefly, liver tissue was retrieved from a −80°C freezer and defrosted, 100 mg of samples was sonicated in 1 mL phosphate-buffered saline to make a 10% homogenate, and then the liver homogenate and serum were treated by sequential sample loading according to the instructions for the detection. Next, the optical density (OD) was measured at 450 nm by using a continuous-wavelength multifunctional enzyme analyzer (Eppendorf, Germany). The content of LPS was determined from standard curves derived from the provided calibrators.

### Histologic, immunohistochemical, and immunofluorescence analyses.

The murine liver and ileum were fixed in 4% paraformaldehyde overnight at 4°C, dehydrated, soaked in xylene, embedded in paraffin in sequence, and then sliced into 4-μm sections. Paraffin sections were dewaxed with xylene and then dehydrated with different concentrations of ethanol. Sections were subjected to H&E staining and immunohistochemical as well as immunofluorescence staining. The following primary antibodies were used for immunohistochemical staining: IL-1β (Bioss, China), IL-6 (Bioss), IFN-γ (Bioss), IL-17A (Bioss), Caspase-1 (ABclonal, China) and NLRP3 (ABclonal). For immunofluorescence on tissues, ZO-1 (Abcam, UK), occludin (Abcam), F4/80 (Abcam), and Alexa Fluor 555 (BBI, China) were used. Furthermore, images were obtained under a microscope (AxioObser Z1, Germany) at a magnification of ×200, and positive results were quantified using ImageJ software (Free Software Foundation Inc., Boston, MA).

### Flow cytometry analysis.

The spleen was removed aseptically and placed on a 200-mesh sieve moistened with PBS. Then the spleen samples were ground using a grinding rod. After the erythrocyte lysate was added, the suspension was centrifuged and the supernatant was discarded. The pellet was washed with PBS, and splenocytes were maintained in 1640 medium containing 10% fetal bovine serum, 1% 100 U/mL penicillin, 100 μg/mL streptomycin. The cells were counted, and the cell concentration was adjusted to 1.0 × 10^7^/mL.

To detect Treg cells, 200 μL of the single cell suspension was stained with APC-CD4 (BD Biosciences, USA), and PE-Foxp3 (BD Biosciences) monoclonal antibodies (MAbs), incubated at room temperature in the dark for 30 min, and analyzed by fluorescence-activated cell sorter (FACS). The data were evaluated using FlowJo software (BD Biosciences).

### Western blot analysis.

Liver tissues were sonicated in ice-cold phosphate-buffered saline (PBS) containing a phosphatase inhibitor cocktail (Beyotime, China) and a protease inhibitor cocktail (Beyotime). Protein concentrations were determined by a quantitative bicinchoninic acid (BCA) protein assay kit (Boster, China). A total of 20 to 30 μg of protein was used for immunoblotting. Primary antibodies against TLR4 (BBI, China), NF-κb (Immuno way USA), IκB (Abcam, UK), P-IκB (Abcam), β-actin (Cell Signaling Technologies [CST], USA), and horseradish peroxidase (HRP)-conjugated goat anti-rabbit secondary antibodies were applied in the study. Next, the electrochemiluminescence (ECL) (Boster) developer was added for imaging and detected using the Tanon imaging system. Finally, the quantification of bands was performed by densitometric analysis using ImageJ software.

### Quantitative real-time PCR.

Total RNA from tissues was extracted using TRIzol (Seven, China) and reverse transcribed into cDNA using the reverse transcription kit (Mei5 Biotechnology, China). The levels of mRNA were measured by real-time PCR with SYBR green reagent (Mei5 Biotechnology) on a QuantStudio 3 system (Applied Biosystems, USA). The expression of individual genes was normalized to the mRNA level of β-actin. The gene-specific PCR primers (all for mouse genes) are as follows: β-actin f, ATCAGCAAGCAGGAGTATG; β-actin r, GGTAGAGGACCACTTTGCTA; ZO-1 f, GCGAACAGAAGGAGCGAGAAGAG; ZO-1 r, GCTTTGCGGGCTGACTGGAG; Occludin f, TGGACTTGGAGGCGGCTATGG; and Occludin r, AGGGAAGCGATGAAGCAGAAGGC. The relative amounts of the different mRNAs were quantified with the threshold cycle (ΔΔ*CT*) method, and the fold change ratio was calculated and expressed as mean ± SEM.

### 16S rRNA microbiome sequencing.

Fecal samples were freshly collected from individual mice, flash frozen in −198°C, and then stored at −80°C until processing. DNA in the feces was extracted with a DNA extraction kit (Qiagen, USA). Beyond that step, the forward primer (F-5′-CCTACGGGRSGCAGCAG-3′) and the reverse primer (R-5′-GGACTACVVGGGTA TCTAATC-3′) were adopted to amplify the V3-V4 regions of the 16S rRNA gene whose amplicon and sequencing were performed as described previously by Kang et al. (53). After that process, the resulting sequences were completed, quality filtered, clustered, and taxonomically assigned on the basis of 97% similarity level against the Ribosomal Database Project (RDP) by using the QIIME software package (version 1.9.1; Knight Lab, San Diego, CA, USA). Statistical analyses were performed, and bacterial abundance and diversity were calculated (54). Phylogenetic Investigation of Communities by Reconstruction of Unobserved States (PICRUSt) based on OTUs was employed to predict the abundances of functional categories using Kyoto Encyclopedia of Genes and Genomes (KEGG) orthology (KO), and at the same time, BugBase was adopted to forecast bacterial composition based on the sequencing results (54).

### Statistical analysis.

The numeric results are shown as the means ± SEM. All data meet the assumptions of the tests (e.g., normal distribution). One-way analysis of variance (ANOVA) was used for comparison among the different groups. When ANOVA was significant, *post hoc* testing of differences between groups was performed using the least significant difference (LSD) test. The exact value of n within figures was indicated in figure legends. For every figure, statistical tests are justified as appropriate. Here, it should be noted that a *P* value of 0.05 was considered statistically significant.

### Data availability.

The data that support the findings of this study are available from the corresponding author, upon reasonable request.
