# Supplementary figures and images for: A Novel Synbiotic Alleviates Autoimmune Hepatitis by Modulating the Gut Microbiota-Liver Axis and Inhibiting the Hepatic TLR4/NF-κB/NLRP3 Signaling Pathway
Source: mSystems. 2023 Feb 16;8(2):e01127-22. doi: 10.1128/msystems.01127-22 (PMC10134874; doi:10.1128/msystems.01127-22)

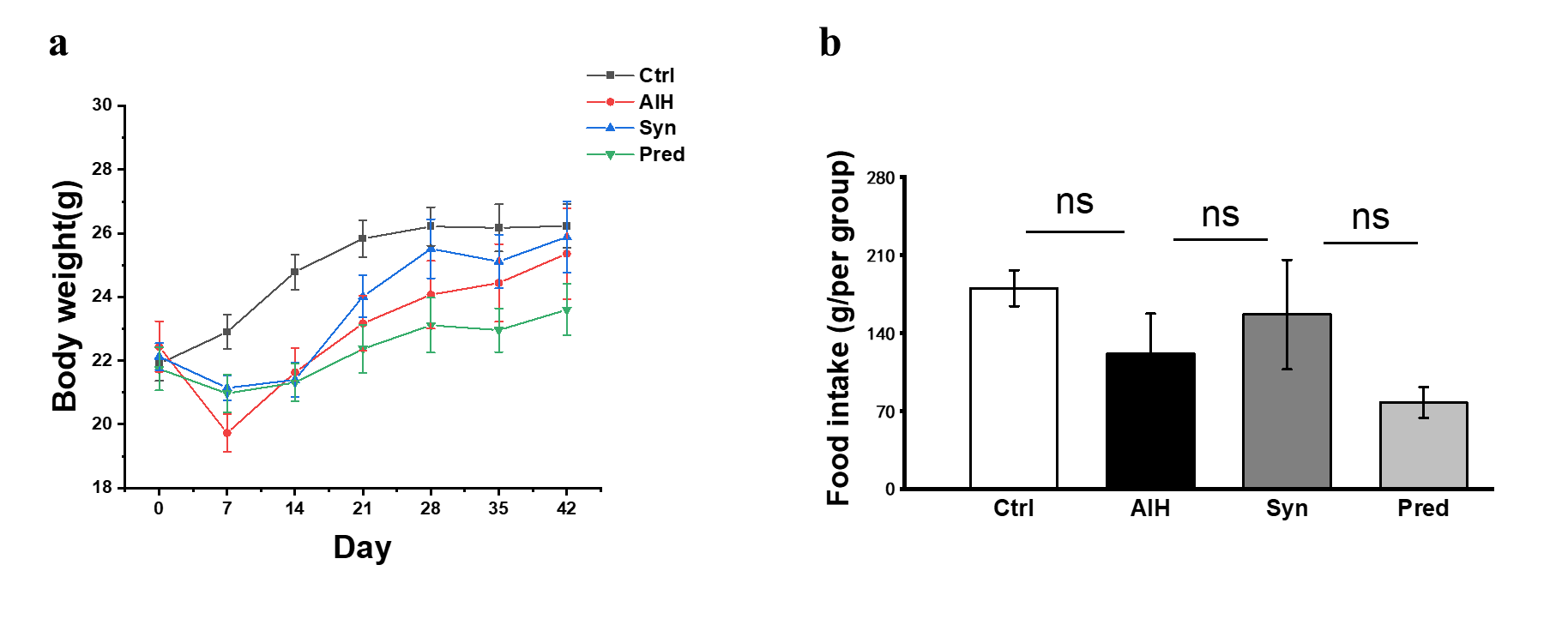

Supplement: FIG S1 [file msystems.01127-22-s0001.tif]

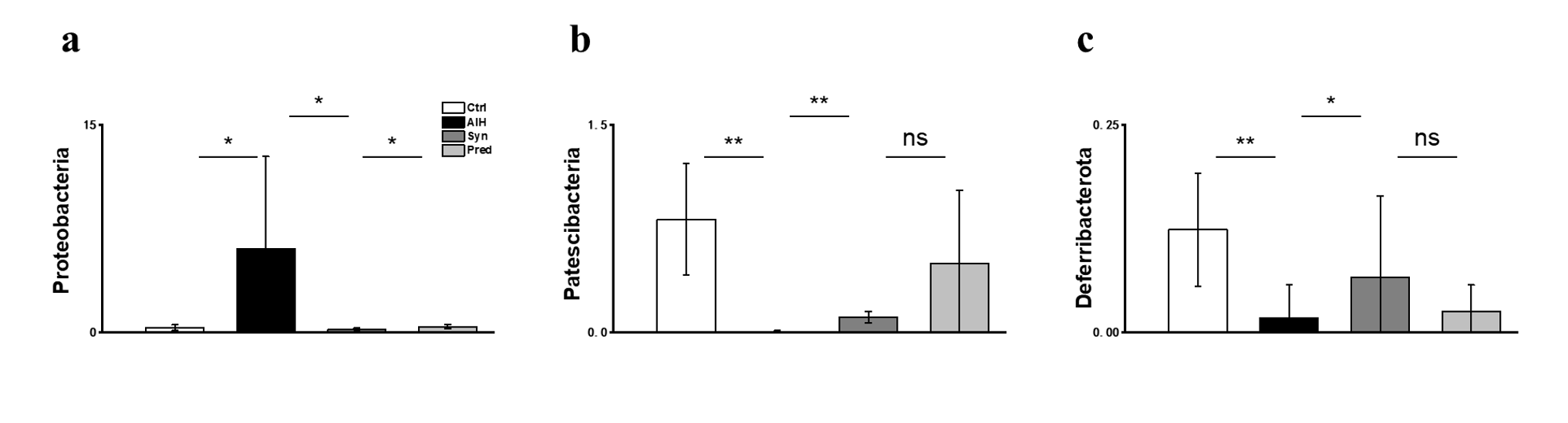

Supplement: FIG S2 [file msystems.01127-22-s0002.tif]

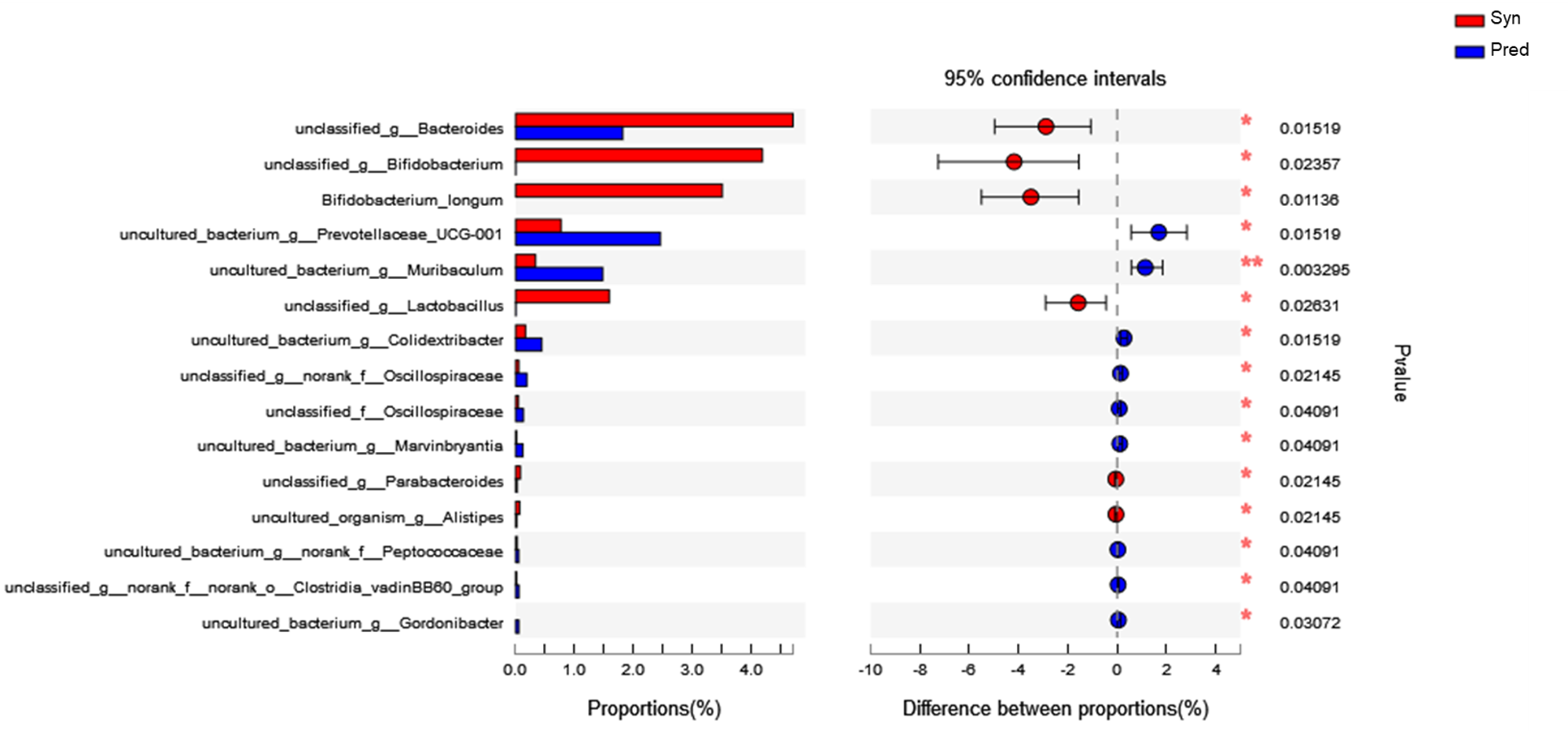

Supplement: FIG S3 [file msystems.01127-22-s0003.tif]

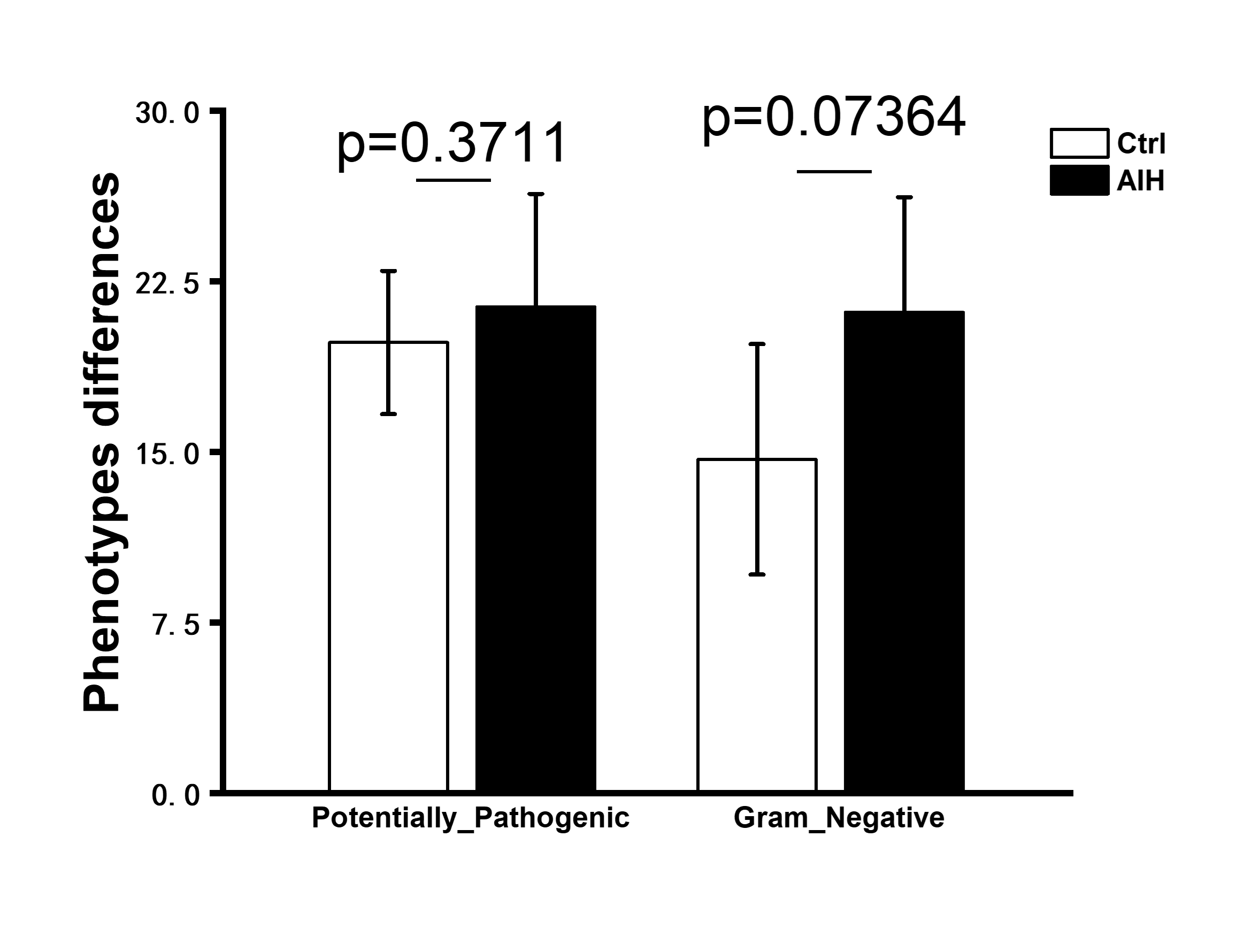

Supplement: FIG S5 [file msystems.01127-22-s0005.tif]

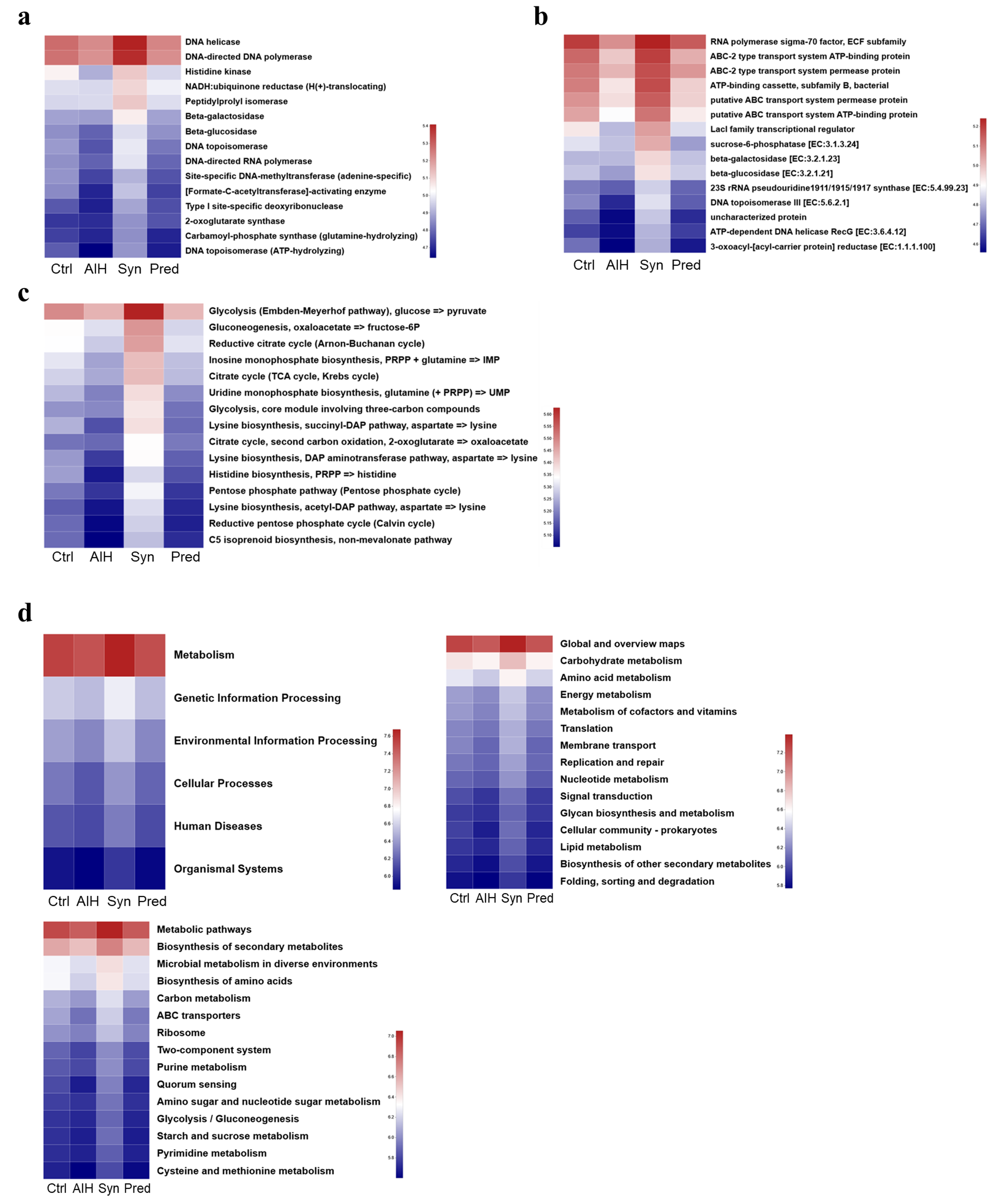

Supplement: FIG S4 [file msystems.01127-22-s0004.tif]

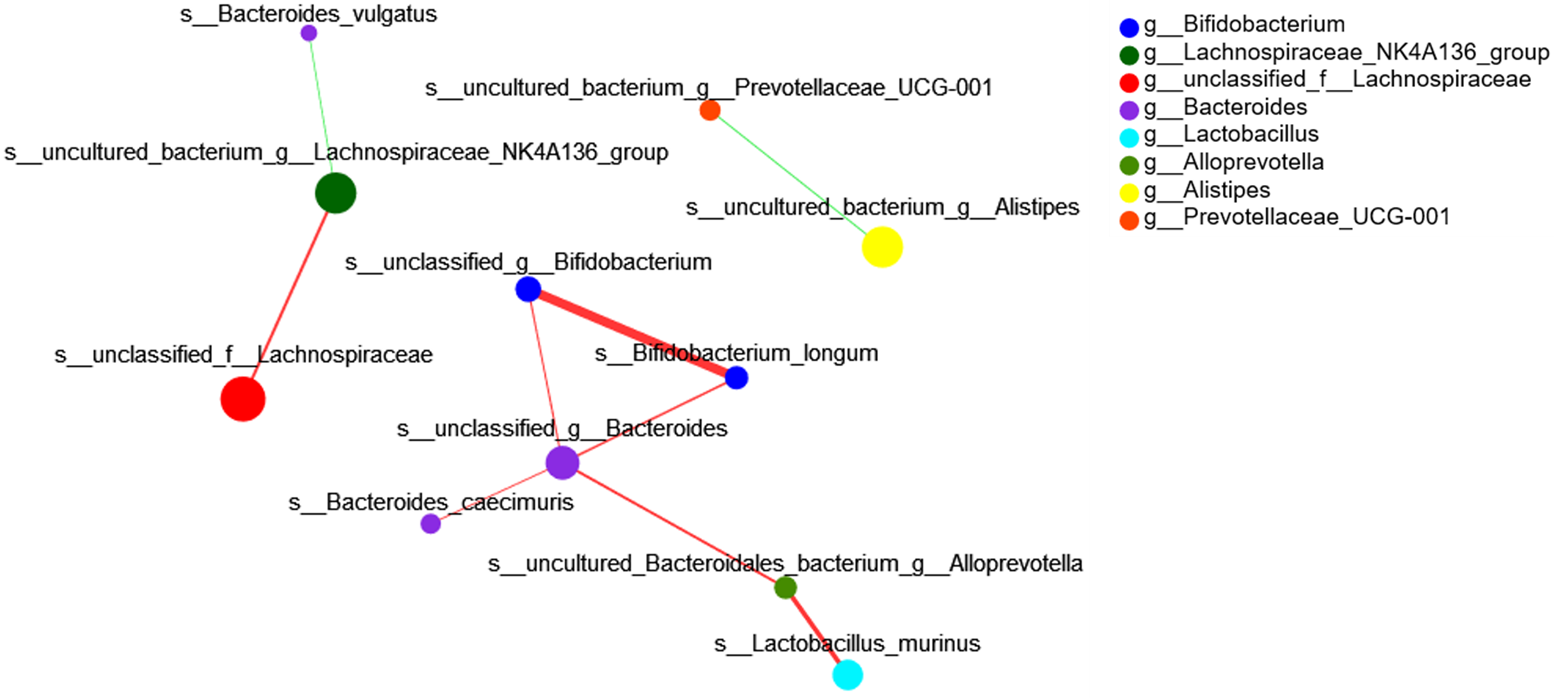

Supplement: FIG S6 [file msystems.01127-22-s0006.tif]

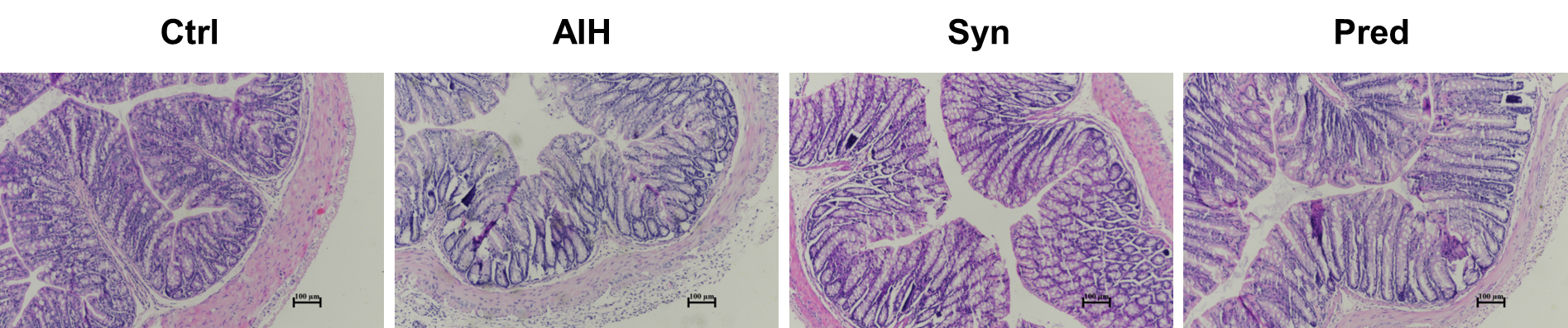

Supplement: FIG S7 [file msystems.01127-22-s0007.tif]
